# The Bearded Vulture as an accumulator of historical remains: Insights for future ecological and biocultural studies

**DOI:** 10.1002/ecy.70191

**Published:** 2025-09-11

**Authors:** Antoni Margalida, Sergio Couto, Sergio O. Pinedo, José María Gil‐Sánchez, Lucía Agudo Pérez, Ana B. Marín‐Arroyo

**Affiliations:** ^1^ Institute for Game and Wildlife Research IREC (CSIC‐UCLM‐JCCM) Ciudad Real Spain; ^2^ Pyrenean Institute of Ecology (CSIC) Jaca Spain; ^3^ Laboratory of Biocultural Archaeology MEMOLab, Departamento Historia Medieval Universidad de Granada, Edf. Josefina Castro Vizoso Granada Spain; ^4^ Consejería de Desarrollo Sostenible de Castilla‐La Mancha Toledo Spain; ^5^ Departamento de Zoología, Facultad de Ciencias Universidad de Granada Granada Spain; ^6^ Grupo I+D+i EvoAdapta (Evolución Humana y Adaptaciones durante la Prehistoria), Departamento de Ciencias Históricas University of Cantabria Santander Spain

**Keywords:** ancient nests, ethnobiology, long‐term occupancy, taphonomy, vulture

Territorial raptors typically occupy their territories over long periods of time. Since usable nest sites are valuable resources for raptors and serve a signal function for conspecifics of habitat quality (Jiménez‐Franco et al., 2014; Newton, 1979; Sergio et al., 2011), long‐term nest reuse over decades and centuries can be usual for some species. For example, C‐14 analyses of fecal material accumulated in a Gyrfalcon (*Falco rusticolus*) eyrie in Greenland demonstrated that it had been occupied for at least 2500 years (Burham et al., 2009). Similarly, an analysis of the twigs in a Golden Eagle (*Aquila chrysaetos*) nest in western North America showed that it had been constructed more than 500 years ago (Ellis et al., 2009). The records of 19th and early 20th century ornithologists also record cases of long‐term nest occupancy. Based on information obtained from the literature, Ramírez et al. (2016) documented the long‐term occupancy (1900–2015) of an Egyptian Vulture (*Neophron percnopterus*) nest in the Canary Islands.

Most vulture species breed on cliffs and carry food to the nest for their chicks in their crop. However, some species such as the Bearded Vulture (*Gypaetus barbatus*) and the Egyptian Vulture mainly use their beaks to carry food, and Bearded Vultures also sometimes use their talons. In the case of cliff‐nesting species, their well‐protected eyries situated in cliff caves, rock shelters, or on cornices allow the accumulation of food remains in the eyries, as well as natural or anthropogenic material used to build the nest (Ellis et al., 2009; Sanchis Serra et al., 2014).

The Bearded Vulture is the most threatened vulture in Europe, with only 309 breeding pairs, 144 of which are in the Pyrenees. However, during the 19th century, the species was distributed in all of the mountainous areas of the Iberian Peninsula and other European mountains. The Bearded Vulture is a cliff‐nesting species characterized by a specialized osteophagous diet (Margalida, Bertran, & Heredia, 2009; Margalida, Sánchez‐Zapata, et al., 2009) that generally uses protected nesting sites such as cliff caves. Its nest sites are characterized by having microclimatic conditions that allow both the accumulated bone remains delivered to the nest to feed the chick and the material used to build the nest to remain in good condition. Pieces of cloth, string, and other anthropogenic manufactured material used to cover the nest bowl for thermoregulatory purposes during incubation are regularly observed in contemporary nests (A. Margalida, personal observation). Feeding ecology can be studied by examination of the accumulated bone, feather, skin, and hair remains in nests (Margalida et al., 2007; Sanz et al., 2025) as well as the occurrence of anthropogenic material. The study of the material preserved in caves housing ancient Bearded Vulture nests can therefore provide interesting information not only about the feeding ecology of the species but also about historical ethnographic and biocultural conditions.

Between 2008 and 2014, we carried out intense research focusing on more than 50 well‐preserved historical Bearded Vulture nests in parts of southern Spain, where the species became extinct around 70–130 years ago (Hiraldo et al., 1979; Margalida, Bertran, & Heredia, 2009; Margalida, Sánchez‐Zapata, et al., 2009; Appendix S1: Table S1). A total of 12 nests were examined, and the remains were identified and analyzed layer by layer, following established archaeological stratigraphic methods.

Here, we describe the materials found in these ancient Bearded Vulture nests, to show the species' long‐term reuse of nests and relevance as an accumulator of various remains. The stratigraphic approach, following archaeological procedures and C‐14 analyses, allowed us to document the age of the nests and some of the material carried to it, providing interesting historical and socioecological information for future research.

Visits to 12 old Bearded Vulture nests (Figure 1) in southern Spain allowed the recovery of a total of 2483 remains (Appendix S1: Table S1), including 2117 bone remains, 43 eggshell remains, 25 items manufactured from esparto grass (*Macrochloa tenacissima*), 86 hooves, 72 leather remains, 11 hair remains, and 129 cloth remains. Comparing biological versus anthropogenic remains (Figures 2 and 3), 2117 remains were related to diet (bone remains) or reproductive processes (43 eggshell fragments) and the remainder (9.1%) comprised 226 anthropogenic remains probably used during nest building. Curiously, we found a crossbow bolt (not dated, Figure 2B) that the birds may have used as nest building material (i.e., in place of a branch) or picked up from the remains of a dead prey item (e.g., a medium‐size wild ungulate) delivered to the nest to feed the chick.

**FIGURE 1:**
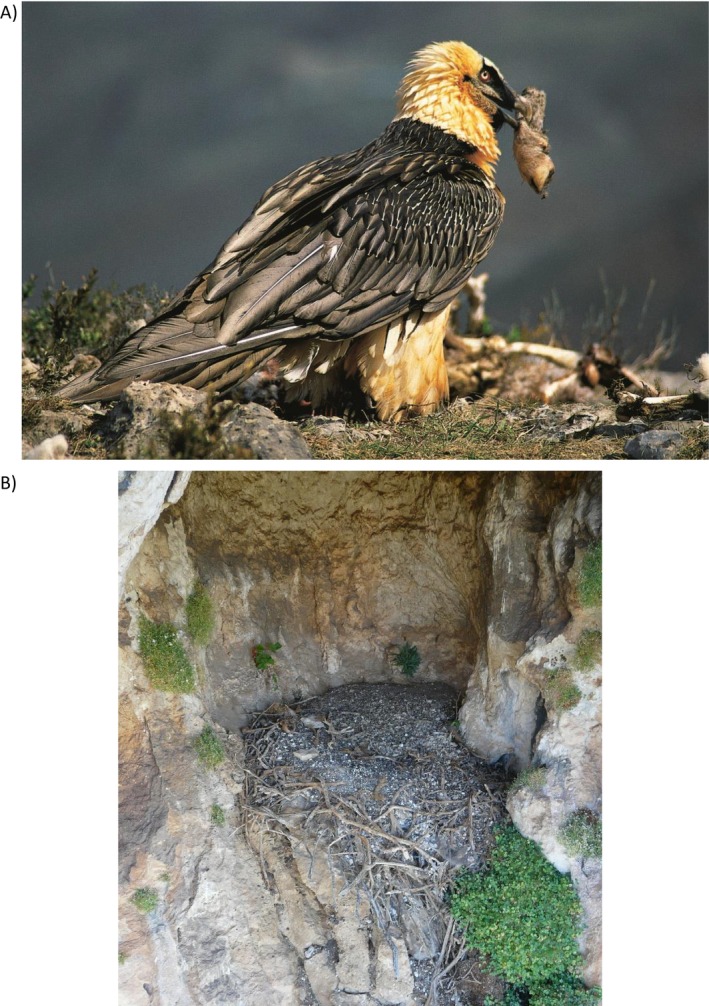
Bearded Vultures typically carry food and nest‐building material to the nest site. (A) An adult Bearded Vulture with a sheep extremity before carrying it to the nest. (B) An example of an ancient Bearded Vulture nest examined, occupied by this species over centuries and easily identified by the outstanding abundance of anthropogenic items made of esparto grass and, typically, solidified white droppings. Photographs: (A) Antoni Margalida; (B) Sergio Couto.

**FIGURE 2:**
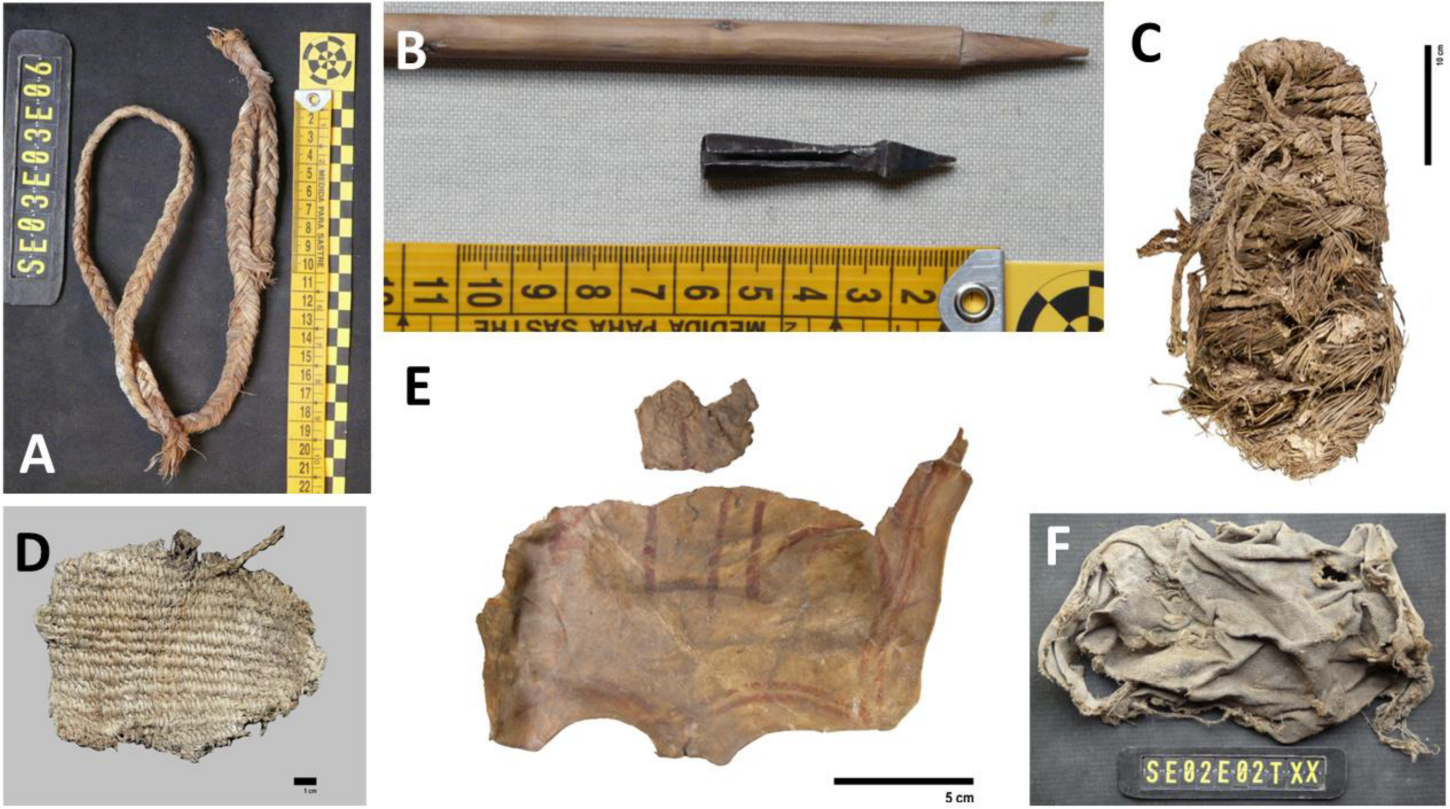
A collection of handcrafted materials found in ancient Bearded Vulture nests. (A) Part of an esparto grass slingshot. (B) A detail of a crossbow bolt and its wooden lance. (C) *Agobía* (Sierra Nevada, Granada), a rough footwear made of several species of grass and twigs, C‐14 dated at 674 ± 22 years Before Present (ETH‐138982). *Agobías* typically lasted for a few days of wear and were continuously repaired and replaced by hand by the wearer. (D) A basketry fragment C‐14 dated at 151 ± 22 years Before Present (ETH‐138980). (E) A piece of sheep leather C‐14 dated at 651 ± 22 years Before Present (ETH‐138981) with red lines drawn, and (F) a piece of fabric. Scale bars are in centimeters. Photographs: Sergio Couto (A, B, D, and F) and Lucía Agudo Pérez (C and E).

**FIGURE 3:**
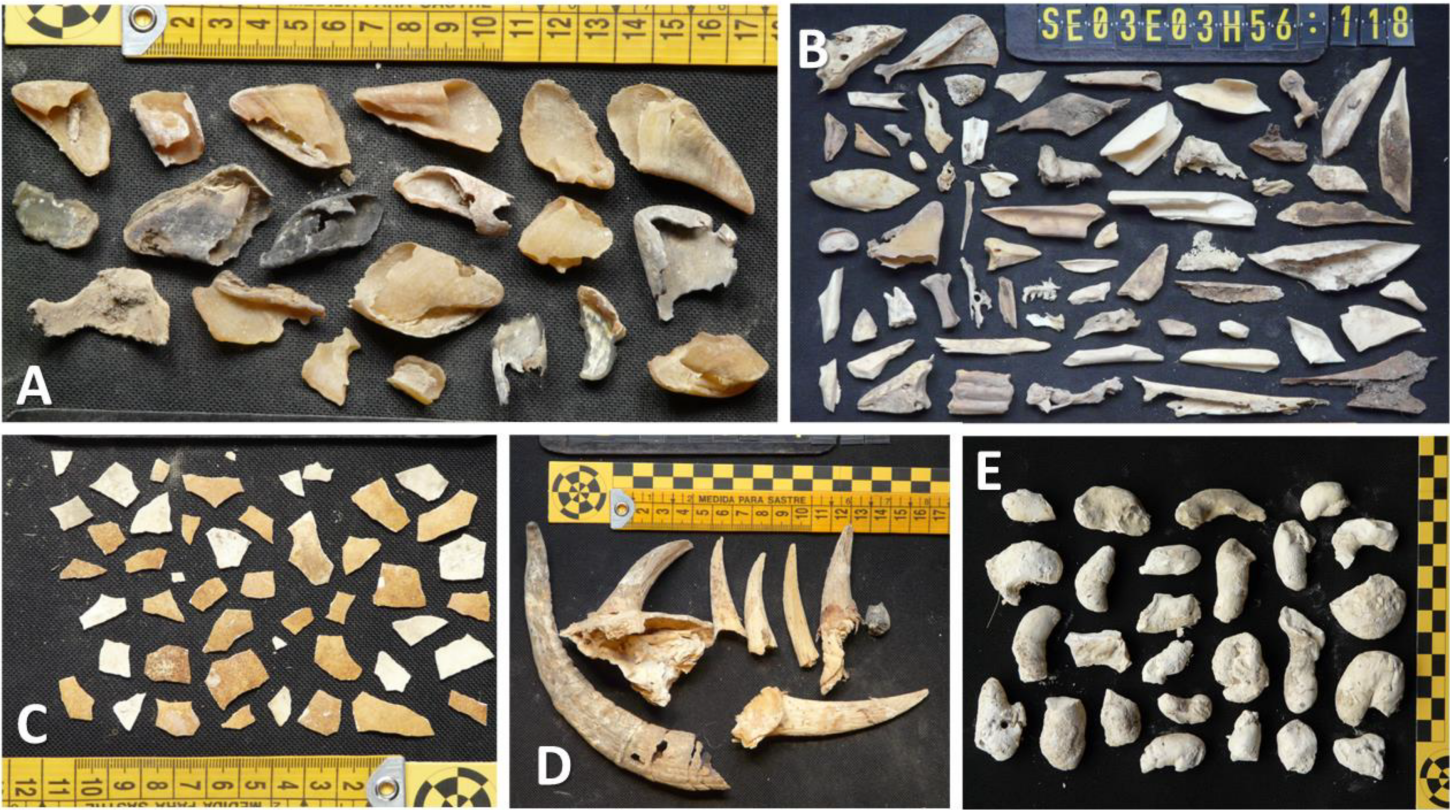
Some items discovered in different ancient Bearded Vulture nests. (A) Hooves typical of ungulate limb remains. (B) Diverse mammal and bird bones and teeth found in the same nest, some partially digested. (C) The remains of Bearded Vulture eggshells, with the typical orange coloration due to the iron oxide applied by the adult birds to their breast feathers. (D) Several goat horns found in the same ancient nest. (E) Typical solid Bearded Vulture droppings accumulated in their ancient nests. Photographs: Sergio Couto.

Initial C‐14 analyses achieved at the Laboratory of Ion Beam Physics (Switzerland) were made on some of the items found in two of the nests (Figure 2). A basketry fragment was found in the upper layer 1 of Nest 02 (Figure 2D). This was dated to 151 ± 22 years Before Present (ETH‐138980), indicating that it was brought to the nest during the late 18th century. A complete sandal made of esparto grass cord was found in the superficial layer of Nest 03 (Figure 2C) and was dated at 674 ± 22 years Before Present (ETH‐138982), corresponding to the late 13th century. Layer 2 of the same nest contained a fragment of ochre‐painted sheep leather (Figure 2E; Appendix S1: Figure S1) (confirmed as sheep by proteomic analysis using ZooMS, Ebsen et al., 2019), dated at 651 ± 22 years Before Present (ETH‐138981). These two C‐14 dates confirmed that the initial layers of Nest 03 were created five centuries earlier than Nest 02 despite their close proximity.

The Bearded Vulture is an accumulator species and has been recognized as a major taphonomic agent (Marín‐Arroyo et al., 2009; Marín‐Arroyo & Margalida, 2012; Robert & Vigne, 2002), behavior which has also been documented for the Egyptian Vulture (Lloveras et al., 2014). The items found in ancient Bearded Vulture nests were mainly ungulate bones, which not only provide relevant information about the dietary habits of the species since medieval times (Margalida & Marín‐Arroyo, 2013) but also provide indirect information on the abundance and distribution of wild vertebrate species inhabiting the area, including animal–human interactions. Therefore, from an ecological perspective, the stratigraphical approach (see Hiemstra et al., 2025) can provide information about temporal changes in the trophic spectrum, past environment, and the wild and domestic species present. Nest accumulations along elevation gradients represent a powerful tool for investigating avian ecology, biodiversity trends, and environmental change. More interestingly, the abundant and well‐preserved anthropogenic elements brought to the nests, such as the extraordinary historical manufactured items made of esparto grass: such as *alpargatas* (esparto sandals), ropes, basketry, horse tacks, and slingshots, have an ethnographic interest. These artifacts can gain significance when considered alongside nest altitude, which influences the availability of remains and the type of ecological zone represented. Extraordinary findings were made, such as several ancient *agobías* (rough footwear made of several species of grass and twigs) and the crossbow bolt (Figure 2B). Similar sandals and basketry artifacts related to the first farming communities were identified related to the Neolithic occupation of Los Murciélagos cave in nearby Granada (35 km away). Some of the nests studied (Martínez‐Sevilla et al., 2023) contained other basketry artifacts similar to those found in Coves de Santa Maira in Valencia (Aura Tortosa et al., 2020). All of these remains attest to the use of plant fibers in the Mediterranean region of the Iberian Peninsula to make a wide variety of artifacts from the Epipaleolithic period, around 12,000 years ago. Accumulators of historical remains, across varying altitudinal and climatic gradients, can thus offer a robust comparative framework for examining the long‐term co‐evolution of ecosystems and human practices, reflecting technological development and shifts in material culture. This opens up future research initiatives to analyze the accumulated anthropogenic contents of ancient Bearded Vulture eyries, as well as other transporting and accumulating species such as the Egyptian Vulture (Lloveras et al., 2014; Sanchis Serra et al., 2014). Thanks to the solidity of Bearded Vulture nest structures and their locations in the western Mediterranean, generally in protected places such as caves and rock shelters (Margalida & Bertran, 2000) with relatively stable temperature and low humidity conditions, they have acted as natural museums, conserving historical material in good condition. This basic historical data and that collected on feeding habits and nest‐site selection provide quality information on the habitat characteristics and food species' selection of this species several centuries ago. In addition, the egg‐shell remains discovered (Figure 3C) allow comparative toxicological studies using contemporary and museum samples (Demarchi et al., 2019) to examine the evidence related to pesticide load and the local extinction history of the Bearded Vulture (Hernández et al., 2018). This information is of the utmost importance for the recovery of the species at the European level, regarding, for example, the species' potential distribution and selection of suitable release sites, or to prioritize habitat conservation efforts.

In addition, these findings have wider relevance for other disciplines, such as ethnobiology and archaeology. For example, the pollen, animal bones, and tree twigs and branches found in ancient nests could provide the basis for chronological and past environmental reconstructions, while the ethnographic and archaeological material could inform studies of ancient human artifacts and provide information on the biocultural heritage of specific mountain areas. From an archaeological point of view, Bearded Vultures as accumulators of bone and human artifacts in northern Iberian caves have provided insights into the prehistoric human groups who also lived there (Marín‐Arroyo et al., 2009; Marín‐Arroyo & Margalida, 2012). Stratigraphical studies of the old nests of accumulator species such as Bearded and Egyptian Vultures (Lloveras et al., 2014; Sanz et al., 2025) provide new taphonomic and interdisciplinary information about regional ecology as well as the local ethnographic, historical, and biocultural conditions. Thus, the Bearded Vulture could be regarded as a bioindicator of exceptional value for long‐term ecosystem monitoring and interdisciplinary research.

## AUTHOR CONTRIBUTIONS

Antoni Margalida, Sergio Couto, Sergio O. Pinedo, and Ana B. Marín‐Arroyo conceptualized the project and established the methodology. Sergio Couto, Sergio O. Pinedo, and José María Gil‐Sánchez carried out fieldwork. Sergio Couto and Lucía Agudo Pérez achieved the classifications on the materials from the nests, and Lucía Agudo Pérez studied them. Ana B. Marín‐Arroyo funded the C14 and proteomic analysis. Antoni Margalida and Ana B. Marín‐Arroyo led the writing of the manuscript, and all coauthors contributed to the final version of the manuscript.

## CONFLICT OF INTEREST STATEMENT

The authors declare no conflicts of interest.

## Supporting information


Appendix S1.
