# Supplementary material for: The Bearded Vulture as an accumulator of historical remains: Insights for future ecological and biocultural studies
Source: Ecology. 2025 Sep 11;106(9):e70191. doi: 10.1002/ecy.70191 (PMC12423732; doi:10.1002/ecy.70191)
Supplement: Supplementary file 1 — Appendix S1. [file ECY-106-e70191-s001.pdf]

APPENDIX S1: SUPPLEMENTARY MATERIAL

The Bearded Vulture as an accumulator of historical remains: Insights for future ecological and biocultural studies

Antoni Margalida, Sergio Couto, Sergio O. Pinedo, José María Gil-Sánchez, Lucía Agudo Pérez, Ana B. Marín-Arroyo

Ecology

Table S1. Diversity of biological and anthropogenic remains collected in 12 bearded vulture historical nests in the region of Granada and Albacete (southern Spain).

Nest	Animal bones	Egg-shell	Esparto grass	Hooves	Leather	Hair	Cloth
NE01	146	3	Various	0	3	0	0
NE02	98	2	5	33	8	5	3
NE03	105	5	Various	1	9	0	Various
NE04	209	4	Various	25	13	0	Various
NE05	21	5	Various	0	5	0	1
NE06	77	3	Various	2	1	6	10
NE07	229	7	20	1	7	0	15
NE08	81	2	Various	0	0	0	Various
SE01	233	3	Various	Various	2	0	25
SE02	101	2	Various	Various	2	0	16
SE03	560	5	Various	Various	14	0	9
SE10	257	2	Various	24	8	Various	50
**Total**	**2117**	**43**	**25**	**86**	**72**	**11**	**129**
**Average**	**176.4**	**3.6**	**2.1**	**7.2**	**6.0**	**0.9**	**10.8**

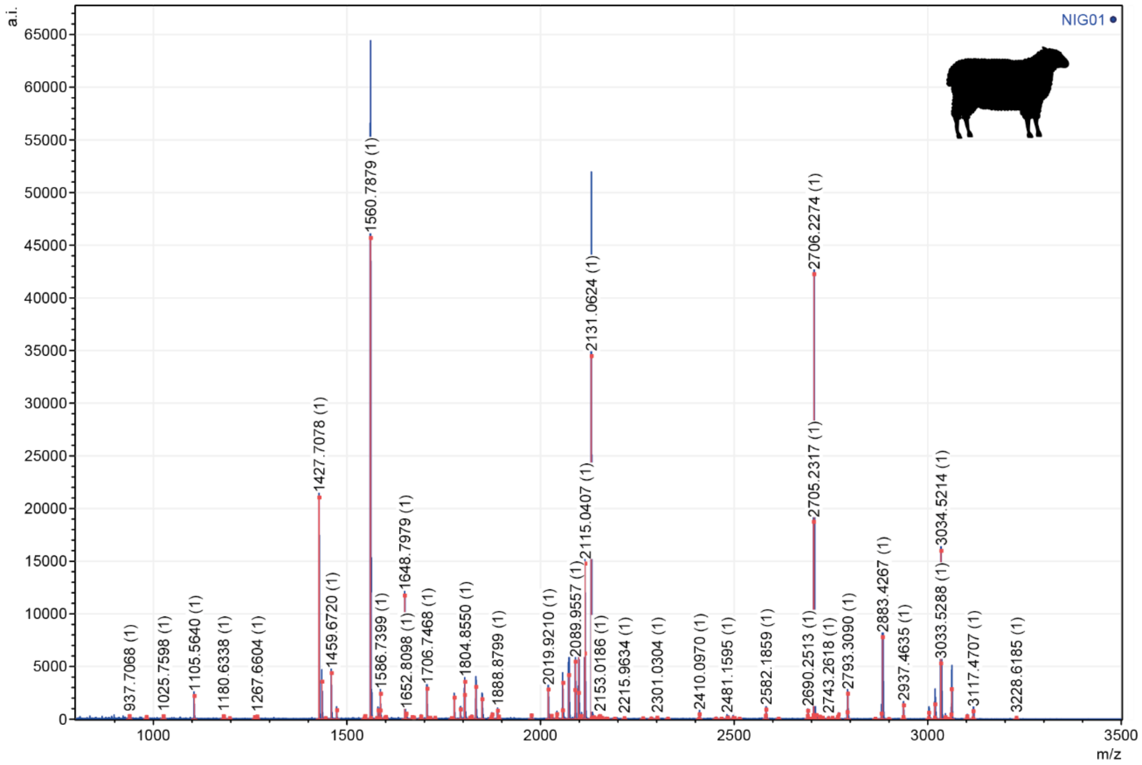

Figure S1. Leather ochre painted sample found in the nest 2, analyzed in the program mMass v.5.5.0 after ZooMS protocol. The results show spectra indicating that the leather belongs to a sheep (*Ovis aries*). Taxonomic identification was made in open source program MMass v. 5.5.0. This program and its documentation are Copyright 2005-2013 by Martin Strohalm (Strohalm 2023). This program is free software that can downloaded under the terms of the GNU General Public License as published by the Free Software Foundation. Available in: https://github.com/chhh/mmass?tab=readme-ov-file

Ovis aries silhouette was downloaded from https://www.phylopic.org/ with CC0 1.0 Universal Public Domain Dedication license.

References

Strohalm, M. 2023. *MMass: A Free Mass Spectrometry Tool* (Version 5.5.0) [Computer software]. MMass Development Team. http://www.mmass.org
